# Trans-articular chondrosarcoma grade 2 of proximal phalanx resulting in its fracture along with destruction of middle phalanx of 2^nd^ toe right foot: a case report and review of the literature

**DOI:** 10.4076/1757-1626-2-7488

**Published:** 2009-07-06

**Authors:** Sheikh Irfan Bashir, Rajesh Gupta, Haris Nazir Khan, Rayees Ahmed, Ashraf Mohd, Abdul Q Salaria

**Affiliations:** Postgraduate Boys Hostel Room No 112 B, Government Medical College Bakshi Nager, Jammu, Jammu and KashmirIndia

## Abstract

Foot is an unusual site for chondrosarcoma and involvement of phalanges is extremely rare. We report a case of grade 2 chondrosarcoma of proximal phalanx resulting in its fracture along with transarticular extension to the middle phalanx of the 2^nd^ toe of right foot in a 62 year old female. The patient presented with 1 and ½ year history of pain and swelling in right 2^nd^ toe. X-ray showed presence of expanding lytic lesion with amorphous calcification along with fracture proximal phalanx. Fine needle aspiration cytology showed some pleomorphs, osteoclasts and some giant cells. We went for amputation of toe without a biopsy. Histopathological examination of specimen showed multiple pleomorphic cells, nuclear atypia, hyperchromasia with some giant cells (grade 2 chondrosarcoma).

## Introduction

Chondrosarcoma constitute about 9% of primary malignancies of bone. It occurs over a broad age range with peak between 40-60 years. It occurs rarely in feet and extremely rare in toes and is more common in males [1,2]. Most patients present with symptoms of pain [3,4]. People with higher - grade tumours i.e. Grade 2 and 3 have pain up to 80% of time [5]. Rarely people will discover fracture in the bone in which they have tumour [1]. In addition to pain they may have swelling; decreased range of motion of the nearby joints. The patient needs to be investigated with investigations like X-Ray of the part. Computed tomography scan gives better delineation of the tumour while bone scan shows increased uptake by the tumour and Magnetic resonance imaging helps by detecting the extent of the bone marrow involvement. As the tumour may metastasize computerized tomography of the chest along with other base line investigations are recommended.

Once the tumour is diagnosed the intermediate grade tumours need wide local resection or amputation e.g. In case of phalanges the resection can be done without biopsy to prevent local tumour contamination. After wide resection the incidence of local recurrence is about 10% and can be treated by repeat wide resection or wide amputation. So primary wide amputation serves as definitive procedure.

## Case presentation

**Figure 1.**
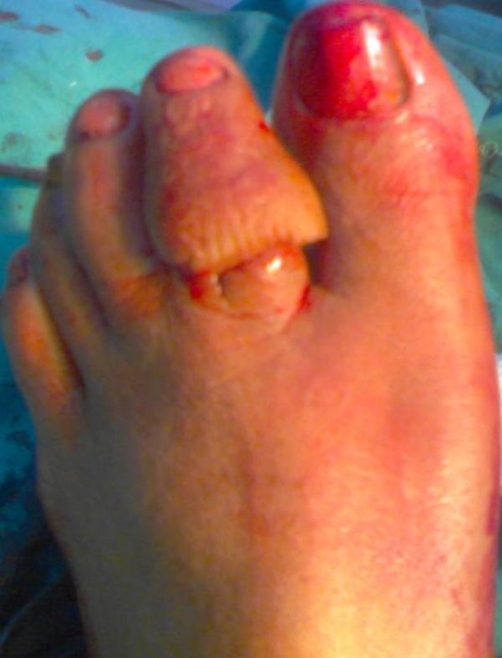
Foot showing the swelling the 2^nd^ toe.

A 62 year old Indian female belonging to lower socioeconomic status presented to the outpatient department with complaints of pain in the 2^nd^ toe of right foot since 1nad ½ year along with swelling of the same toe since 6 months. Pain was dull boring type and remained throughout the day, with no radiation. The swelling was progressively increasing. Patient went to local quacks who prescribed various massages etc. There was no other co-morbid history in the past and no similar complaints in the family. On examination patient had swollen 2^nd^ toe giving it a drum stick appearance (Figure 1). The swelling was firm to hard in consistency tender on palpation with irregular and ill defined margins, movements were painful and restricted at interphalangeal joint but normal at metatarsophalangeal joint.

**Figure 2.**
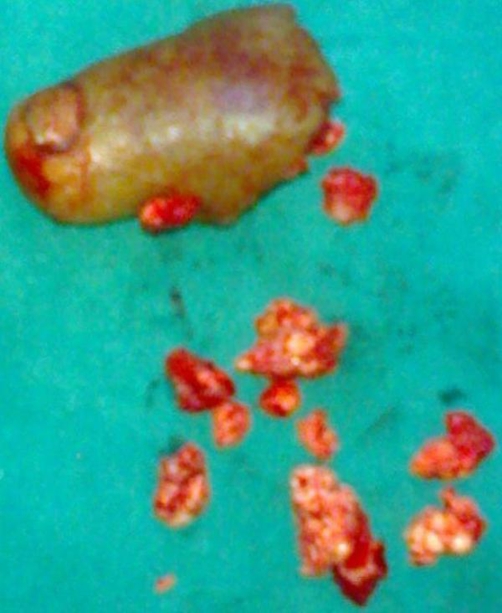
Gross appearance of the tumour.

**Figure 3.**
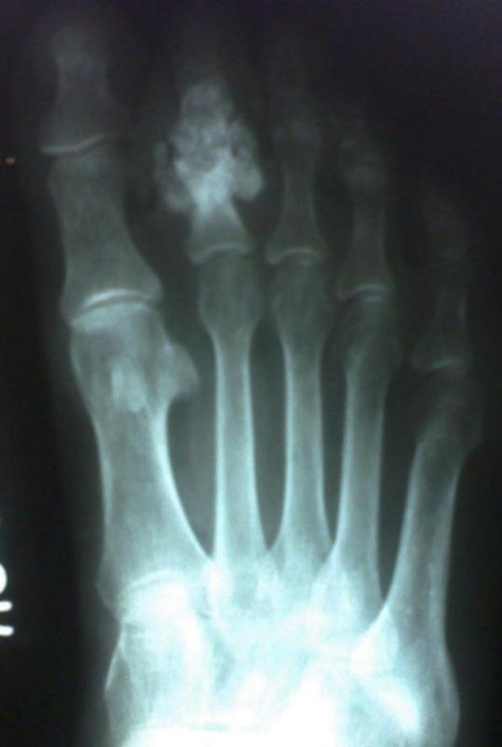
Preoperative radiograph of the foot.

**Figure 4.**
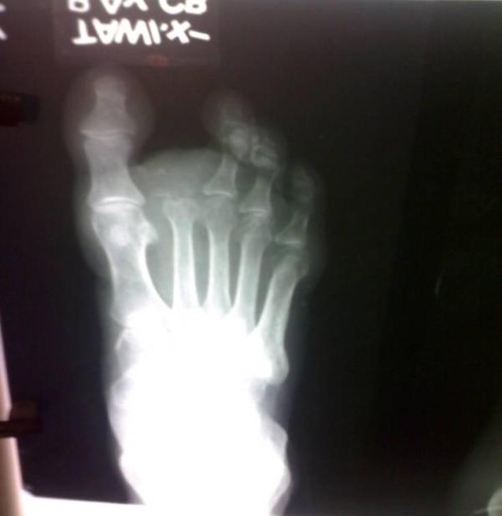
Postoperative radiograph of the foot.

Patient underwent Anterior-Posterior and lateral radiographs which showed fracture of the proximal phalanx along with destruction of the middle phalanx, amorphous calcifications were present along with endosteal scalloping (Figure 3,4). Computerized tomography was not possible because of the financial implications of the patient. Fine needle aspiration cytology showed pleomorphic cells, binucleate cells and certain giant cells. All routine investigations along with chest x-ray were within normal limits. A bold decision of the amputation of the toe was taken after thorough discussion with the patient. The amputated sample was sent for histopathological examination which on gross examination showed marrow cavity occupied by greyish white material eroding the cortex and infiltrating surrounding tissue (Figure 2). Histology showed Anisonucleosis, Hyperchromasia, Bizzare nuclei and giant cells with lobulated chondromatous tissue, confirming it to be the primary chondrosarcoma grade 2 (intermediate grade) a rare variety among the chondrosarcoma itself. Post-operatively patient was put in foot orthosis and patient recovered normally. There was no local or distant recurrence afterwards and patient is still under follow-up.

## Discussion

Chondrosarcoma is a malignant bone tumour containing tumour cells that produce cartilage. This tumour may form de novo as a primary lesion or from malignant transformation of a pre-existing benign condition or cartilage lesion eg. Enchondroma or osteochondroma.

There numerous types of primary chondrosarcoma including conventional intramedullary, clear cell, juxta-cotical, myxoid, mesenchymal, extra-skeletal and dedifferentiated chondrosarcomas. The primary conventional intramedullary chondrosarcoma also known as central or medullary chondrosarcoma is more commonly seen in adults 30 years and older, with most frequently affected sites being the pelvis and long bones, especially the femur and the humerus in up to 65% of the cases, with short tubular bones of hand and feet being rarer sites (1-4% of all cases), with less than 200 cases reported [2,4,6]. The local recurrence rate of grade 3 chondrosarcoma is 47%, while the reported rate of distant metastasis is 10-50% for grade 2 lesions and 50-71% for grade 3 lesions. Metastasis most commonly involve lung, regional lymph nodes and liver. The overall 5-year survival rates for chondrosarcoma are 90-29% (grade3) [2]. Phalangeal chondrosarcoma behaves as a locally aggressive tumour and in contrast to chondrosarcomas located elsewhere, rarely metastasises.

Chondrosarcomas are categorized as central, peripheral, or juxtacotical (periosteal) leisions depending in their osseous location. Central chondrosarcomas are intramedullary in origin, and large enough to erode cortex and invade the surrounding soft tissue [2]. Bovee et al [4] reported 35 cases of chondrosarcoma of the phalanx, with the range of patient age at the time of diagnosis being 21-87 years. There was a slight female predominance. Occurrence in the hand was noted more commonly than in the foot, with proximal phalanx affected most often. Epiphyseal involvement is rare and joint involvement is reported to be even less common [9]. We have found only one case report of trans-articular extension of a primary bone neoplasm [10]. Histological grading of conventional intramedullary chondrosarcoma correlates with clinical behaviour and prognosis. A three-grade system is most commonly used:

Grade 1 chondrosarcomas – low grade, with a predominantly chondroid stroma, with distinction of grade 1 chondrosarcoma from enchondroma often difficult [2].

Grade 2 chondrosarcomas – intermediate grade, have less chondroid matrix and are correspondingly more cellular. Necrosis may be seen [2].

Grade 3 chondrosarcomas – high grade, show greater cellularity and nuclear pleomorphism than grade 2 tumours. Chondroid matrix is sparse or absent. Foci of necrosis are seen and are frequently extensive [2].

The treatment for high grade chodrosarcomas is wide/radical resection or amputation. For lesions in expandable location, primary wide resection or amputation without biopsy may be indicated to decrease the chance of tumour contamination [11,12].

## Abbreviations

None.
